# Analysis of normal levels of free glycosaminoglycans in urine and plasma in adults

**DOI:** 10.1016/j.jbc.2022.101575

**Published:** 2022-01-08

**Authors:** Sinisa Bratulic, Angelo Limeta, Francesca Maccari, Fabio Galeotti, Nicola Volpi, Max Levin, Jens Nielsen, Francesco Gatto

**Affiliations:** 1 Department of Biology and Biological Engineering, Chalmers University of Technology, Göteborg, Sweden; 2 Department of Life Sciences, University of Modena and Reggio Emilia, Modena, Italy; 3 Department of Molecular and Clinical Medicine/Wallenberg Laboratory, Institute of Medicine, Sahlgrenska Academy, University of Gothenburg, Gothenburg, Sweden; 4 Department of Oncology, Sahlgrenska Academy, University of Gothenburg, Göteborg, Sweden; 5 Department of Oncology, Sahlgrenska University Hospital, Gothenburg, Sweden; 6 BioInnovation Institute, Copenhagen N, Denmark

**Keywords:** glycosaminoglycans, chondroitin sulfate, heparan sulfate, hyaluronic acid, GAGome, reference intervals, biomarkers, CS, chondroitin sulfate, GAGs, glycosaminoglycans, GLP, good laboratory practice, HA, hyaluronic acid, HS, heparan sulfate, PSA, prostate-specific antigen, UHPLC-MS/MS, ultra-high-performance liquid chromatography coupled with triple-quadrupole tandem mass spectrometry

## Abstract

Plasma and urine glycosaminoglycans (GAGs) are long, linear sulfated polysaccharides that have been proposed as potential noninvasive biomarkers for several diseases. However, owing to the analytical complexity associated with the measurement of GAG concentration and disaccharide composition (the so-called GAGome), a reference study of the normal healthy GAGome is currently missing. Here, we prospectively enrolled 308 healthy adults and analyzed their free GAGomes in urine and plasma using a standardized ultra-high-performance liquid chromatography coupled with triple-quadrupole tandem mass spectrometry method together with comprehensive demographic and blood chemistry biomarker data. Of 25 blood chemistry biomarkers, we mainly observed weak correlations between the free GAGome and creatinine in urine and hemoglobin or erythrocyte counts in plasma. We found a higher free GAGome concentration – but not a more diverse composition - in males. Partitioned by gender, we also established reference intervals for all detectable free GAGome features in urine and plasma. Finally, we carried out a transference analysis in healthy individuals from two distinct geographical sites, including data from the Lifelines Cohort Study, which validated the reference intervals in urine. Our study is the first large-scale determination of normal free GAGomes reference intervals in plasma and urine and represents a critical resource for future physiology and biomarker research.

Glycosaminoglycans (GAGs) are a family of long, linear polysaccharides consisting of repeating disaccharide units (1). Different classes of GAGs have been characterized. In humans, the most prevalent classes are chondroitin sulfate (CS) [(→3)-β-D-GalNAc(1→4)-β-D-GlcA or α-L- IdoA(1→], heparan sulfate (HS) [(→4)-α-D-GlcNAc or α-D- GlcNS(1→4)-β-D-GlcA or α-L-IdoA (1→], and hyaluronic acid (HA) [(→3)-β-D-GlcNAc(1 → 4)-β-D-GlcA(1→] where GalNAc is N-acetylgalactosamine, GlcA is glucuronic acid, IdoA is iduronic acid, GlcNAc is N-acetylglucosamine, and GlcNS is N-sulfoglucosamine. Glucuronic acid can be further modified by sulfation in up to three sites. Chondroitin Sulfate and HS disaccharides can each be further modified with O-sulfo groups, typically in up to three positions in humans. The resulting sulfation motifs confer GAGs highly diverse biological functions that are essential for healthy human development and physiology (2). The panel of GAG motifs resulting from the diversity in structure and concentration of GAGs is collectively referred to as GAGome.

Alterations in the physiological function of GAGs have been associated with several diseases ranging from mucopolysaccharidosis, a group of rare metabolic disorders caused by genetic defects in lysosomal enzymes that degrade GAGs, to complex diseases such as sepsis, rheumatoid arthritis, and cancer (3, 4, 5, 6). Plasma and urine GAGomes have been proposed as promising biomarkers for early noninvasive diagnostics (6). Despite the potential role of GAGs for clinical applications, the measurements of the GAGome has been limited to very small sample sizes (ranging from 3 healthy donors in (7) to 25 in (6)), in predominantly retrospective and selected donors, with different analytical techniques performed within academic laboratories (3, 4, 6, 7, 8, 9). These limitations can be attributed to the historical lack of effective analytical methods until recently (10, 11, 12, 13, 14), which proved hard to standardize and expensive to run. As a result, the GAGome measurements reported in the literature for healthy subjects are widely variable and cannot be consistently used as reference for physiology and biomarker research.

In this study, we took advantage of a standardized analytical method using ultra-high-performance liquid chromatography coupled with triple-quadrupole tandem mass spectrometry (UHPLC-MS/MS) (15) to analyze the urine and plasma protein-free fraction of GAGomes (or free GAGomes, in short). We analyzed the free GAGomes in a good laboratory practice (GLP)-compliant blinded central laboratory in two prospectively enrolled independent cohorts of 308 self-reported healthy subjects with comprehensive demographic and blood chemistry data. We first determined the correlation of free GAGomes with demographic and blood chemistry variables. Next, we established reference intervals for the normal free GAGome in urine and plasma according to accepted guidelines (CLSI EP28-A3c). Finally, we validated the proposed references intervals by transference analysis on two independent cohorts consisting of a total of 140 healthy individuals from two distinct geographical sites.

## Results

### Subject characteristics

We prospectively enrolled two cohorts of self-rated healthy adults with no history nor family history of cancer (except nonmelanoma skin cancer) from one site in Stockholm, Sweden (Cohort 1, *N* = 292 and 2, *N* = 16), for a total of 308 participants (Table 1). Cohort 1 and 2 formed the reference sample group to establish reference intervals.

**Table 1:** Subject characteristics in the reference sample group (Cohort 1 and Cohort 2)

Characteristic	Cohort 1	Cohort 2	Cohort 1 + 2
N	292	16	308
Age	57 (22–78)	43 (27–51)	57 (22–78)
Gender			
Female	183	5	188
Male	109	11	120
Self-rated health			
Moderate	10	0	10
Good	153	5	158
Very good	129	11	140
Blood chemistry biomarkers			
ALAT (μkat/l)	0.36 (0.13–2.44)	0.49 (0.27–1.1)	0.37 (0.13–2.44)
ASAT (μkat/l)	0.41 (0.22–1.65)	0.41 (0.27–0.59)	0.41 (0.22–1.65)
Calcium (mmol/l)	2.39 (2.15–2.72)	2.42 (2.17–2.58)	2.39 (2.15–2.72)
Creatinine (μmol/l)	69 (46–167)	80 (59–102)	69.5 (46–167)
C-reactive protein (mg/l)	1.4 (0.2–54.1)	0.65 (0.27–5.5)	1.3 (0.2–54.1)
Estimated glomerular filtration rate (ml/min/1.73 m^2^)	82.5 (33–90)	90 (76–90)	83 (33–90)
Glycated hemoglobin (mmol/mol)	-	33.5 (28–39)	33.5 (28–39)
HDL (mg/dl)	61.87 (29.78–123.74)	-	61.87 (29.78–123.74)
LDL (mg/dl)	131.48 (46.4–239.75)	-	131.48 (46.4–239.75)
Potassium (mmol/l)	4.2 (3.5–5.2)	4.2 (3.9–4.9)	4.2 (3.5–5.2)
Prostate specific antigen level (ng/ml)	0.8 (0.11–9.8)	0.7 (0.33–1.4)	0.8 (0.11–9.8)
Sodium (mmol/l)	139 (132–145)	140.5 (138–144)	139 (132–145)
Complete blood count			
Hematocrit (%)	0.42 (0.35–0.51)	0.44 (0.37–0.48)	0.42 (0.35–0.51)
Hemoglobin (g/l)	141 (116–178)	145.5 (118–163)	141 (116–178)
Mean corpuscular hemoglobin (pg)	30 (24–37)	30 (27–31)	30 (24–37)
Mean corpuscular hemoglobin concentration (g/l)	336 (304–366)	331 (315–351)	336 (304–366)
Mean corpuscular volume (fl)	90 (81–101)	89.5 (86–94)	90 (81–101)
Absolute neutrophil count (10^9^/l)	3.2 (1–7.7)	-	3.2 (1–7.7)
Basophil count (10^9^/l)	0 (0–0.2)	-	0 (0–0.2)
Eosinophil count (10^9^/l)	0.1 (0–2.8)	-	0.1 (0–2.8)
Erythrocyte count (10^12^/l)	4.6 (3.8–5.8)	4.85 (4.1–5.3)	4.6 (3.8–5.8)
Leukocyte count (10^9^/l)	5.6 (2.8–10.8)	5.75 (3.5–7.7)	5.6 (2.8–10.8)
Lymphocyte count (10^9^/l)	1.7 (0.7–4.5)	-	1.7 (0.7–4.5)
Monocyte count (10^9^/l)	0.4 (0.2–0.9)	-	0.4 (0.2–0.9)
Platelet count (10^9^/l)	261 (137–488)	239 (166–377)	261 (137–488)
Blood chemistry status			
No abnormal values	135	11	146
1–2 abnormal values	134	5	139
>2 abnormal values	23	0	23

Distributions are summarized as median and min-max range in brackets. Missing values were omitted.

Subjects’ characteristics were balanced between cohorts (Table 1). Across cohorts, the median age was 57 years (range: 22–78) with 188 (61%) women and 120 (39%) men. Virtually all subjects self-reported good (51%) or very good (45%) health status. The panel of blood chemistry markers measured in Cohort 1 and 2 was the same, except for HDL/LDL (measured in Cohort 1 only) and glycated hemoglobin (measured in Cohort 2 only). Complete blood counts were available for Cohort 1, whereas Cohort 2 had available measurements for erythrocyte, leukocyte, and platelet counts. Blood chemistry showed normal values for all biomarkers in 47% of the subjects, values outside the reference intervals for up to 2 biomarkers in 45% of the subjects, and more than two abnormal values in 23 subjects (8%). The abnormal blood chemistry values were mostly because of elevated calcium (12.2 % subjects), low sodium (10.4%), low C-reactive protein (7.5%), low estimated glomerular filtration rate (7.2%), and elevated prostate-specific antigen (PSA) (5%). We kept subjects with abnormal values in the reference sample group and performed a sensitivity analysis of the reference values within this subgroup.

The free GAGomes in plasma and urine were measured in a single GLP-compliant blinded laboratory using a standardized kit on UHPLC-MS/MS in all Cohort 1 and 2 subjects (*N* = 308).

### Effect of blood chemistry on the normal free GAGome

We sought to characterize whether other blood chemistry biomarkers were correlated with the normal free GAGome in plasma or urine.

First, we classified subjects in groups indicative of their general health status depending on the number of abnormal values for the blood chemistry biomarkers here tested. Specifically, we grouped the subjects into three groups: 1) no abnormal values (“No abnormal value”, *N* = 146), 2) one or two abnormal values (“1–2 abnormal values”, *N* = 139), or 3) more than two abnormal values (“>2 abnormal values”, *N* = 23). We did not observe any statistical associations between any GAGome feature with any of these groups (Table S1).

Second, we investigated linear correlations between the concentration of each detectable GAGome feature and each of the 25 blood chemistry biomarker level (as a continuous variable) and focused on correlations that reached statistical significance after controlling for multiple testing (*p* < 1.67⋅10^−4^, Bonferroni correction, Fig. S2). In urine, we observed weak to moderate positive correlations (ρ = 0.23–0.30) between multiple GAGome features with creatinine. In addition, the urine 6S CS concentration was positively correlated with hemoglobin, hematocrit, and erythrocyte count (ρ = 0.24–0.25) and negatively correlated with HDL (ρ = –0.22). In plasma, the total CS and 4S CS concentrations were weakly positively correlated with hemoglobin and erythrocyte count (ρ = 0.21–0.28). In addition, plasma 4S CS correlated with hematocrit (ρ = 0.25), whereas 0S CS negatively correlated with HDL at ρ = −0.22, (Fig. S2).

### Effect of age and gender on the normal free GAGome

We sought to identify if the free GAGomes in urine and plasma correlated with age and gender that could partition the reference intervals.

We did not observe any statistically significant association between age (as a continuous variable) and any GAGome feature (Table S2 and Fig. S1).

**Figure 1:**
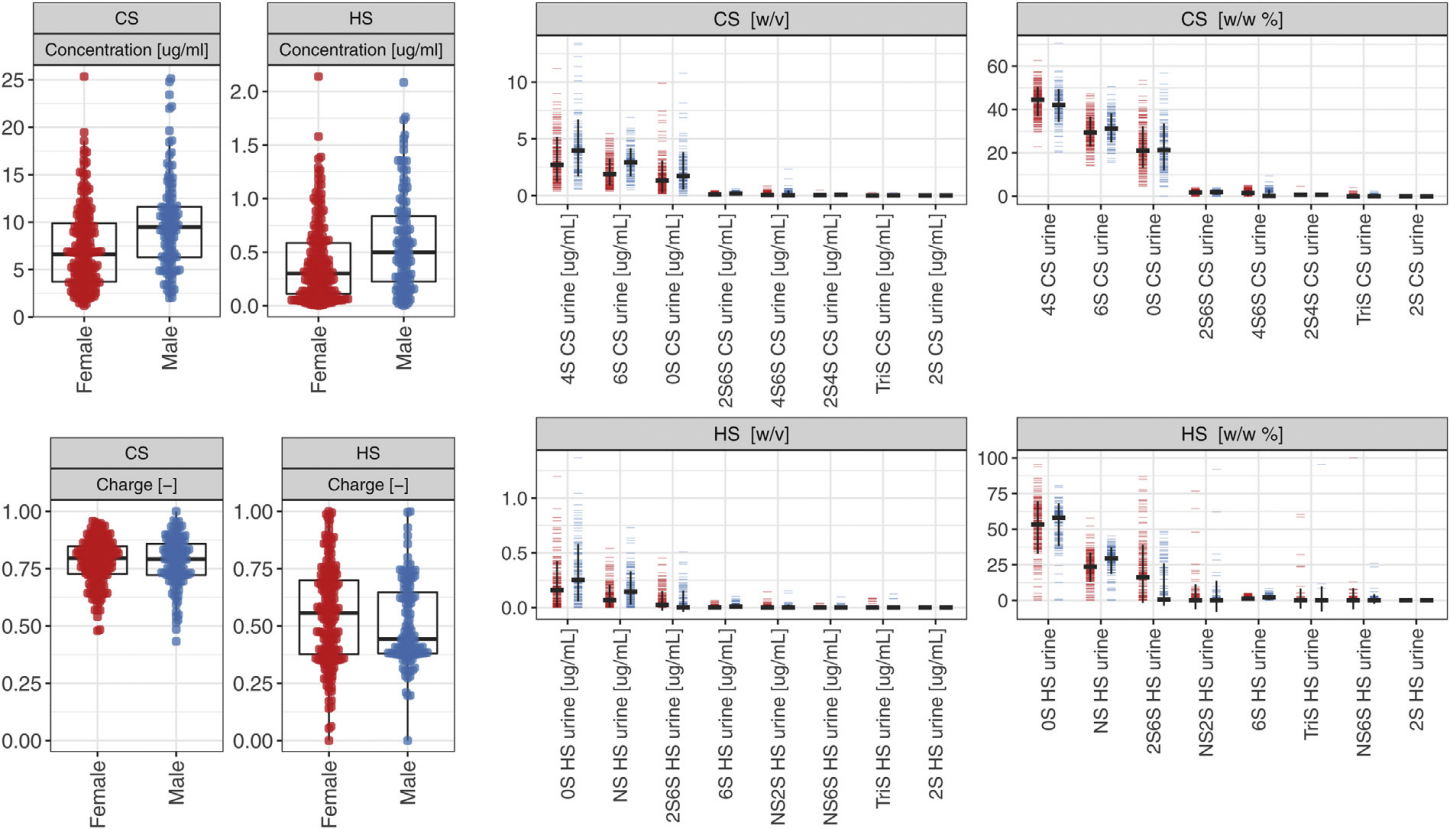
**Totalfreechondroitin sulfate (CS) and heparan sulfate (HS) concentration (μg/ml), charge, and disaccharide concentration (μg/ml) and composition (in mass fraction %) in theurine of thereference sample group by gender (Cohort 1 and 2, *N***_***females***_**= 188 *N***_***males***_**= 120).** The error bars indicate ± 1 SD. Key: *Red* – female, *blue* - male.

**Figure 2:**
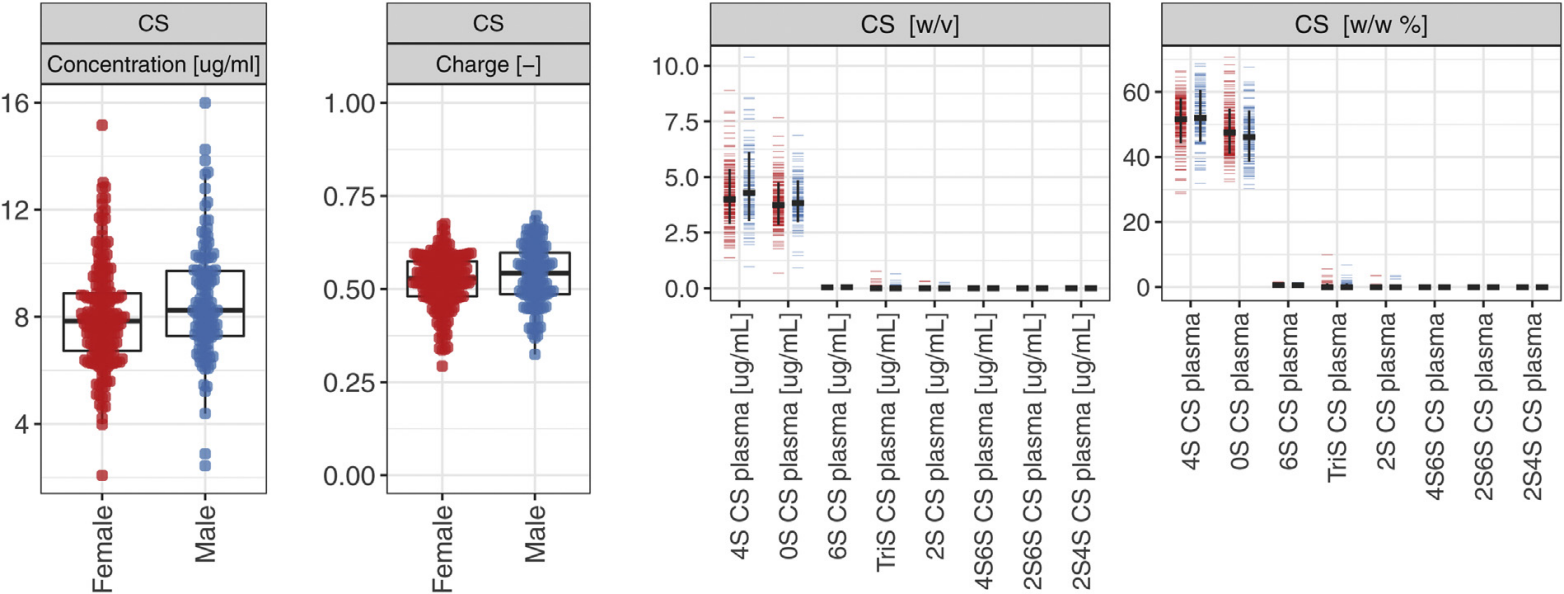
**Totalfreechondroitin sulfate (CS) concentration (μg/ml), charge, and disaccharide concentration (μg/ml) and composition (in mass fraction %) inthe plasma ofthe reference sample group (Cohort 1 and 2).** Free HS was undetectable in plasma. The error bars indicate ± 1 SD. Key: *Red* – female, *blue* - male. HS, heparan sulfate.

We observed statistically significant associations between gender and 10 detectable GAGome features (FDR <0.1, Table S3, Figs. 1 and 2). In plasma, the effect of gender was generally limited to an average 7% increase in total CS and 11% increase of 4S CS concentration in males. The urine of males contained on average 31% to 41% higher concentration for the major CS disaccharides (0S, 4S, 6S, and 2S6S CS), resulting in an average 34% increase in total CS; and an average 50% and 73% higher concentration for the major HS disaccharides (0S and NS, respectively), resulting in an average 47% increase in total HS.

### Reference intervals for the normal free GAGome in urine

Given the effects of gender on the GAGome, we decided to partition reference intervals by gender. We thus defined reference intervals for the free GAGome in urine and plasma across apparently healthy males and females between the ages of 22 and 78.

First, we established reference intervals for the free GAGome in urine after outlier identification and exclusion. For each disaccharide, *bona fide* outliers were identified according to a prespecified procedure and omitted (median % of outliers across CS disaccharides: 2% in males, 0% in females; across HS: 3% in males, 3% in females).

We then established the reference intervals partitioned by gender of all free CS and HS features in urine (Fig. 1 and Table 2).

**Table 2:** Reference intervals of total free chondroitin sulfate (CS) and heparan sulfate (HS) concentration (μg/ml) and disaccharide concentration (μg/ml) and composition (% w/w) in urine by gender

	Female (N = 188)			Male (N = 120)		
	Outliers	Mean	Reference interval	Outliers	Mean	Reference interval
CS						
Concentration						
Total CS [μg/ml]	-	6.99	[1.77–16.04]	-	8.86	[2.62–17.17]
4S CS [μg/ml]	0 (0.0%)	3.19	[0.68–7.42]	2 (2.0%)	4.04	[0.84–10.14]
6S CS [μg/ml]	0 (0.0%)	2.07	[0.54–4.68]	1 (1.0%)	2.90	[0.87–5.67]
0S CS [μg/ml]	2 (1.0%)	1.59	[0.20–5.18]	5 (4.0%)	1.99	[0.36–4.86]
2S6S CS [μg/ml]	0 (0.0%)	0.13	[0.00–0.37]	1 (1.0%)	0.18	[0.00–0.43]
4S6S CS [μg/ml]	9 (5.0%)	0.09	[0.00–0.41]	8 (7.0%)	0.10	[0.00–0.44]
Composition						
4S CS [%]	2 (1.0%)	43.66	[32.00–55.26]	4 (3.0%)	42.10	[29.31–53.73]
6S CS [%]	0 (0.0%)	29.76	[16.38–43.89]	2 (2.0%)	31.19	[19.44–42.51]
0S CS [%]	2 (1.0%)	22.28	[6.57–40.65]	2 (2.0%)	22.62	[6.32–46.47]
2S6S CS [%]	0 (0.0%)	1.73	[0.00–3.31]	0 (0.0%)	1.75	[0.00–3.24]
4S6S CS [%]	0 (0.0%)	1.41	[0.00–4.25]	2 (2.0%)	1.20	[0.00–4.43]
Charge CS	2 (1.0%)	0.78	[0.60–0.93]	1 (1.0%)	0.78	[0.57–0.94]
HS						
Concentration						
Total HS	-	0.25	[0.00–0.68]	-	0.46	[0.00–1.23]
0S HS [μg/ml]	10 (5.0%)	0.18	[0.00–0.55]	2 (2.0%)	0.31	[0.00–0.84]
NS HS [μg/ml]	13 (7.0%)	0.08	[0.00–0.24]	4 (3.0%)	0.16	[0.00–0.42]
Composition						
0S HS [%]	5 (3.0%)	52.57	[18.65–83.45]	5 (4.0%)	55.19	[32.97–73.63]
NS HS [%]	2 (1.0%)	22.96	[1.45–40.31]	8 (7.0%)	29.72	[13.27–41.34]
Charge HS	0 (0.0%)	0.30	[0.06–0.47]	0 (0.0%)	0.35	[0.21–0.44]

Outliers were excluded.

The average total free CS concentration in urine was 8.86 μg/ml in males and 6.99 μg/ml in females. The CS composition in males and females was nearly identical. The three major disaccharides made up 42% and 44% (4S CS), 31% and 30% (6S CS), and 23% and 22% (0S CS) of the CS fraction in urine of males and females, respectively. Of the multi-sulfated CS disaccharides, the 2S6S CS and 4S6S CS cumulatively contributed approximately 3% to the CS fraction in both males and females, whereas 2S4S CS and TriS CS were undetectable. The average urine CS charge was 0.78 for males and females.

The average total free HS concentration in urine was 0.46 μg/ml in males and 0.25 μg/ml in females. The only detectable free HS disaccharides were 0S HS and NS HS. The HS urine composition was 55% 0S HS and 30% NS HS in males and 53% 0S HS and 23% NS HS in females. The average HS charge was 0.35 in males and 0.30 in females.

### Reference intervals for the normal free GAGome in plasma

We repeated the procedure above for the free GAGome in plasma (median % of outliers across CS disaccharides: 0 % in males or 1% in females, Fig. 2 and Table 3). The free HS fraction was largely undetectable in plasma (mean total HS < 0.001 μg/ml), and we therefore omitted it from further analyses.

**Table 3:** Reference intervals of total free chondroitin sulfate (CS) concentration (μg/ml) and disaccharide concentration (μg/ml) and composition (% w/w) in plasma by gender

	Female (N = 188)			Male (N = 120)		
	Outliers	Mean	Reference interval	Outliers	Mean	Reference interval
CS						
Concentration						
Total CS		7.79	[4.91–11.28]		8.52	[5.93–12.78]
4S CS [μg/ml]	6 (3.0%)	4.07	[2.07–6.37]	2 (2.0%)	4.55	[2.25–8.02]
0S CS [μg/ml]	4 (2.0%)	3.78	[2.26–5.48]	4 (3.0%)	3.96	[2.56–5.82]
Composition						
4S CS [%]	2 (1.0%)	51.41	[36.16–62.06]	0 (0.0%)	52.64	[36.64–65.46]
0S CS [%]	1 (1.0%)	48.02	[37.77–64.12]	0 (0.0%)	46.37	[33.92–62.09]
Charge CS	0 (0.0%)	0.52	[0.36–0.62]	0 (0.0%)	0.53	[0.37–0.66]

Free HS was undetectable in plasma. Outliers were excluded.

The average total free CS concentration in plasma was 8.52 μg/ml in males and 7.79 μg/ml in females. The CS composition was nearly identical across genders, 53% and 51% 4S CS and 46% and 48% 0S CS (Table 3) for males and females, respectively. The remaining free CS disaccharides were undetectable. The average plasma CS charge was 0.53 in men and 0.52 in women.

### Transference of reference intervals in an independent population

We validated the transference of the above established reference intervals for each GAGome feature in two independent populations from two distinct geographical sites (Cohort 3 and 4) by determining the free GAGomes in urine and plasma in Cohort 3 (N = 110, 60 males and 50 females) and Cohort 4 (N = 30, 15 males and 15 females). The average age was 59 years old (range 30–84) for Cohort 3 and 45 years old (range 22–66) for Cohort 4. We observed that Cohort 3 and 4 had largely similar GAGome measurements. Therefore, we opted to carry out the transference analysis in a group combining Cohort 3 and Cohort 4, partitioned by gender (*N* = 140, 65 females and 75 males, see Tables S4 and S5 for transference analyses on separate cohorts).

Across all free GAGome features in urine, we excluded 0 to 9.2% (median = 1.5%) outliers in females and 0 to 12% (median = 3.3%) in males. Across all free GAGome features in plasma, we excluded 0 to 4.6% (median = 1.5%) outliers in females and 0 to 1.3% (median = 0%) in males. We next determined the percentage of values outside the established reference limits for each urine and plasma GAGome feature, where <5% was considered acceptable for transference validation.

In urine, we observed that the transference of reference intervals was validated in both genders for the concentration of all detectable free GAGome features (Table 4). The total urine CS was outside the reference interval in 5.6% samples, whereas all samples had total HS within the reference interval. As regards composition, we observed a shift toward a higher 4S CS % (mean in reference sample group: 44% in females, 42% in males; in transference group: 50% in females, 47% in males) and concomitantly other smaller shifts toward lower composition values for the remaining CS disaccharides.

**Table 4:** Transference of reference intervals of free chondroitin sulfate (CS) and heparan sulfate (HS) in urine in an independent population (Cohorts 3 and 4)

	Female (N = 65)					Male (N = 75)				
	N	Mean	Reference interval	Cohort range	Outside reference interval	N	Mean	Reference interval	Cohort range	Outside reference interval
CS										
Concentration										
Total CS [μg/ml]	62	6.2	1.8–16.0	2.3–18.8	2 (3.2%)	72	8.7	2.6–17.2	2.5–26.7	4 (5.6%)
4S CS [μg/ml]	65	3.1	0.7–7.4	1.0–10.2	2 (3.1%)	75	4.1	0.8–10.1	1.4–14.0	2 (2.7%)
6S CS [μg/ml]	65	1.6	0.5–4.7	0.7–6.9	1 (1.5%)	74	2.2	0.9–5.7	0.7–7.3	3 (4.1%)
0S CS [μg/ml]	62	1.2	0.2–5.2	0.3–4.9	0 (0.0%)	73	1.9	0.4–4.9	0.5–5.2	2 (2.7%)
2S6S CS [μg/ml]	64	0.1	0.0–0.4	0.0–0.4	2 (3.1%)	70	0.2	0.0–0.4	0.0–0.4	1 (1.4%)
4S6S CS [μg/ml]	65	0.1	0.0–0.4	0.0–0.4	0 (0.0%)	70	0.2	0.0–0.4	0.0–0.4	0 (0.0%)
Composition										
4S CS [%]	60	49.4	32.0–55.3	32.4–61.8	13 (21.7%)	72	46.9	29.3–53.7	35.4–57.4	10 (13.9%)
6S CS [%]	62	25.4	16.4–43.9	14.2–33.2	2 (3.2%)	73	25.5	19.4–42.5	17.1–33.7	5 (6.8%)
0S CS [%]	64	19.6	6.6–40.6	5.5–44.1	3 (4.7%)	75	22.2	6.3–46.5	6.9–50.6	1 (1.3%)
2S6S CS [%]	64	2.0	0.0–3.3	0.5–3.4	2 (3.1%)	68	2.0	0.0–3.2	0.6–3.6	2 (2.9%)
4S6S CS [%]	65	1.9	0.0–4.2	0.0–4.6	2 (3.1%)	75	2.0	0.0–4.4	0.0–4.8	1 (1.3%)
Charge CS	64	0.8	0.6–0.9	0.6–0.9	6 (9.4%)	74	0.8	0.6–0.9	0.6–0.9	0 (0.0%)
HS										
Concentration										
Total HS [μg/ml]	59	0.3	0.0–0.7	0.0–0.7	1 (1.7%)	66	0.3	0.0–1.2	0.0–0.8	0 (0.0%)
0S HS [μg/ml]	61	0.2	0.0–0.6	0.0–0.7	2 (3.3%)	70	0.2	0.0–0.8	0.0–0.8	0 (0.0%)
NS HS [μg/ml]	61	0.1	0.0–0.2	0.0–0.2	1 (1.6%)	68	0.1	0.0–0.4	0.0–0.2	2 (2.9%)
Composition										
0S HS [%]	65	55.5	18.6–83.5	6.4–80.6	3 (4.6%)	75	52.7	33.0–73.6	2.3–74.7	9 (12.0%)
NS HS [%]	62	22.6	1.4–40.3	8.8–30.7	0 (0.0%)	71	23.4	13.3–41.3	10.9–34.9	6 (8.5%)
Charge HS	65	0.5	0.1–0.5	0.2–1.0	33 (50.8%)	75	0.5	0.2–0.4	0.3–1.0	41 (54.7%)

Outliers were excluded.

In plasma, we could not validate the transference of reference intervals for either of the two detectable free GAGome features in plasma (0S CS and 4S CS) because 5.3 to 28.6% of the transference group had increased concentration (Table 5). The discrepancy was less pronounced for composition, where 14.7 to 23.1% had values outside the reference intervals.

**Table 5:** Transference of reference intervals of free chondroitin sulfate (CS) in plasma in an independent population (Cohorts 3 and 4)

	Female (N = 65)					Male (N = 75)				
	N	Mean	Reference interval	Cohort range	Outside reference interval	N	Mean	Reference interval	Cohort range	Outside reference interval
CS										
Concentration										
Total CS [μg/ml]	62	10.0	4.9–11.3	6.7–14.9	18 (29.0%)	74	10.1	5.9–12.8	6.7–15.9	4 (5.4%)
4S CS [μg/ml]	63	5.6	2.1–6.4	3.2–8.5	18 (28.6%)	75	5.5	2.3–8.0	2.9–11.0	4 (5.3%)
0S CS [μg/ml]	64	4.4	2.3–5.5	2.6–8.5	7 (10.9%)	74	4.5	2.6–5.8	2.4–8.2	12 (16.2%)
Composition										
4S CS [%]	65	56.3	36.2–62.1	30.0–72.3	14 (21.5%)	75	54.6	36.6–65.5	33.7–72.8	11 (14.7%)
0S CS [%]	64	42.8	37.8–64.1	26.9–59.1	14 (21.9%)	75	44.7	33.9–62.1	26.4–65.9	11 (14.7%)
Charge CS	65	0.6	0.4–0.6	0.3–0.7	15 (23.1%)	75	0.6	0.4–0.7	0.3–0.7	12 (16.0%)

Outliers were excluded.

## Discussion

In this study, we established reference intervals for the free GAGome in urine and plasma in a large adult healthy population by taking advantage of a standardized high throughput UHPLC-MS/MS method (15). In addition, the extensive demographic and biochemical characterization of subjects allowed us to assess novel correlations with the free GAGome.

We found that no free GAGome feature showed any notable differences with respect to age in adults. Although this finding in urine agree with previous studies analyzing total — as opposed to free — GAGomes (16), it contradicts similar studies in plasma, where an increase with age was reported in males only (17). In contrast to age, we found that the concentrations of several CS and HS disaccharides were higher in males than females with a larger effect in urine than plasma. Previous studies noted gender differences in total GAGomes, although in the opposite directions for both urine and plasma (16, 17). The origin of these discrepancies remains to be ascertained, but we speculate that they could be attributed to the focus on the total rather than the free GAGome fraction in previous studies, as well as older analytical techniques for GAG measurements and smaller sample sizes.

We also observed that free GAGomes in urine appeared independent of other blood chemistry biomarkers, including markers of inflammation, glucose metabolism, and liver functions — underscoring that they may reflect an independent physiological state in adults. However, it should be noted that molecular weight and chain length of GAGs are known to play an important role in modulating biological processes such as inflammation - as in the case of high-molecular and low-molecular weight HA (18), as well as HS and CS (19, 20). The analytical method used herein did not provide measurements on molecular weight and chain length, which may reflect the lack of correlation with, for example, inflammation markers. The only blood chemistry biomarker that correlated moderately with free GAGomes, and specifically urine GAGomes, was serum creatinine. We believe that this correlation indicates that urine-free GAGomes depend on the extent of urine excretion rather than being informative of renal function. Thus, we speculate that normalization by urinary creatinine could render free GAGome measurements in urine more robust. In plasma, free CS showed weak correlations with markers related to erythrocytes, particularly 4S CS. This association has been previously described for platelets where their activation can lead to rapid increases of circulating 4S CS (21), but not in the context of other blood cells such as erythrocytes.

We observed that the reference intervals established for free GAGomes were remarkably tight in the reference sample group. In plasma, each free GAGome feature deviated by ∼75% at most from the mean. Similarly, in the urine, each feature deviated by a maximum of ∼3-fold from the mean. These findings suggest that free GAGomes in urine and plasma have stable and predictable levels in a healthy adult. This conclusion appears corroborated by the transference analysis that validated all reference intervals established for urine in independent adult samples from two different geographical sites. Even among the free GAGome features that failed to validate the reference intervals, chiefly in plasma, the deviation from the reference sample group was limited. For example, the mean plasma 4S CS concentration was 4.1 μg/ml in the female reference sample group and 5.6 μg/ml in the transference group, that is 36% higher. In comparison, the reference interval for the platelet count in the reference sample group spans 137 to 488•10^9^ l^−1^, a 3-fold difference between the reference limits. Considering this, we speculate that if a substantial deviation of a free GAGome feature from the here reported reference interval is observed in an adult, then this deviation may be more indicative of a disease state rather than physiological variability. This makes free GAGomes suitable candidates for biomarker studies.

In conclusion, this study established and validated reference intervals for free GAGomes in urine and plasma in the largest adult population to date. As such, we believe that this study represents a critical resource for physiology and biomarker research using free GAGomes in biofluids.

## Experimental procedures

### Study design

This study was designed and conducted in compliance with the CLSI. Defining, Establishing, and Verifying Reference Intervals in the Clinical Laboratory; Approved Guideline—Third Edition. The collection of specimens was planned prospectively with *a priori* criteria for population sampling. Human studies reported here abided by the Declaration of Helsinki principles. The present study was approved by the Ethical Committee in Göteborg, Sweden (Etikprövningsmyndigheten) (#737–17 and #198–16).

### Reference sample group population

This study prospectively enrolled self-rated healthy adult subjects in one site in Sweden forming two independent cohorts. Cohort 1 and 2 were used to form the reference sample group and they were both enrolled at Sabbatsberg Hospital, Stockholm between May 2018 and December 2019. The inclusion criteria were as follows: adults between 21 and 78 years old; at least moderate self-rated health; no history of cancer (except nonmalignant skin cancer); no family history of cancer (first-degree relative); fit to undergo protocol procedures. The exclusion criteria were as follows: abnormal PSA value in the last 5 years. Eligible participants were identified among volunteers based on a questionnaire by trained research nurses. Participants in each cohort formed a consecutive series. Ethylenediaminetetraacetic acid-plasma and spot-urine samples were collected in one single visit. Blood samples were also obtained by venipuncture from participants in Cohort 1 and 2 to evaluate laboratory biomarkers informative of the general health status of the subject, including the complete blood count, the concentration of electrolytes (sodium, potassium, and calcium), ALAT, ASAT, C-reactive protein, PSA, glycated hemoglobin (Cohort 2 only), LDL, and HDL (Cohort 1 only). The subjects with abnormal values were referred for clinical examination but were otherwise retained in the study.

### Transference population

The transference analysis was performed on two cohorts (Cohorts 3 and 4) representative of healthy adults from two distinct and external geographical sites. Cohort 3 used retrospectively archived specimens from the Lifelines Cohort study (22). The inclusion criteria were as follows: adults older than 18 years old; self-reported healthy. The exclusion criteria were as follows: diagnosis of cancer within 18 months from sampling visit. Lifelines is a multi-disciplinary prospective population-based cohort study examining in a unique three-generation design the health and health-related behaviors of 167,729 persons living in the North of the Netherlands. It uses a broad range of investigative procedures in assessing the biomedical, socio-demographic, behavioral, physical, and psychological factors which contribute to the health and disease of the general population, with a special focus on multi-morbidity and complex genetics. Cohort 4 was enrolled prospectively at Sahlgrenska University Hospital, Göteborg, Sweden. The inclusion criteria were as follows: adults older than 18 years old; self-reported healthy. The exclusion criteria were as follows: none.

### Glycosaminoglycan measurements

Urine was collected at room temperature in a polypropylene cup. Ethylenediaminetetraacetic acid-plasma was collected through venipuncture in a vacuette at room temperature and next subjected to centrifugation (1100–1300*g*, 20 min at room temperature in Cohort 1 and 2500*g* 15 min at 4 °C in Cohort 2) within 15 min. The samples could be stored refrigerated (4 °C) for 12 h before transfer to a freezer (−20 °C in Cohort 1 and −70 °C in Cohort 2). Shipment was performed at the same temperature as storage.

Sample preparation was performed in a single blinded GLP-compliant laboratory using the MIRAM Free Glycosaminoglycan Kit (Elypta AB) to extract GAGs from urine and plasma samples. Glycosaminoglycan detection and quantification was obtained through UHPLC-MS/MS in accordance with the instruction for use in of the kit (15). Briefly, the method relied on the enzymatic digestion of the protein-free fraction of full-length GAGs into disaccharides using *Chondroitinase ABC* and *Heparinase I-II-III* (11). The released disaccharides were subsequently labeled using 2-aminoacridone and injected into an ultra-high-performance liquid chromatography coupled with electrospray ionization triple-quadrupole mass spectrometry system (Waters Acquity I-class Plus Xevo TQ-S micro) for separation and detection. The peaks of the 17 disaccharides were acquired using multiple reaction monitoring with all multiple reaction monitoring parameters prespecified as per kit instructions (15).

Laboratory measurements of the protein-free fraction of GAGomes (free GAGomes) included the absolute concentration in μg/ml of CS, HS, HA disaccharides, resulting in 17 independently measured features. Specifically, we quantified eight CS disaccharides (0S CS, 2S CS, 6S CS, 4S CS, 2S6S CS, 2S4S CS, 4S6S CS, and TriS CS) and eight HS disaccharides (0S HS, 2S HS, 6S HS, NS HS, NS6S HS, NS2S HS, 2S6S HS, TriS HS) — corresponding to different sulfation patterns of CS and HS — and one HA disaccharide. A measured GAGome feature was considered detectable in a fluid (plasma or urine) if its mean concentration across all samples was >0.1 μg ml^−1^ (15).

The detectable free GAGome was further used to calculate additional dependent features, including the total amount of CS and HS. The absolute concentration in μg/ml of total CS and HS were calculated as the sums of the absolute concentrations of the eight individually measured CS and HS disaccharides, respectively. Furthermore, we calculated the CS and HS composition (as mass fractions of individual detectable CS and HS disaccharides) and the CS and HS charge (as the ratio of all sulfated CS or HS disaccharides weighted by their charge to all CS or HS disaccharides weighted by their charge).

### Statistical analysis

Before all analyses, GAGome features were transformed using the Box-Cox transformation to identify and exclude outliers. Reference intervals were established for each free GAGome feature in urine and plasma using a simple nonparametric method after outlier exclusion. The lower and upper reference limits (reference intervals) for each GAGome feature was estimated as the 2.5^th^ and 97.5^th^ percentiles of the distribution of measured values in the reference population, respectively.

The correlation between each GAGome feature and each clinical (*e.g.*, age) or biochemical (*e.g.*, LDL) variable was calculated by univariable linear regression of a given GAGome feature as response variable on a given clinical or biochemical variable as explanatory variable. We computed the statistical significance of each correlation using a likelihood ratio test versus an intercept-only regression model. Multiple hypothesis testing was adjusted using the Benjamini–Hochberg or Bonferroni corrections depending on if clinical or biochemical variables were tested, with *FDR* values <0.1 and *p*-values < 1.67⋅10^−4^ considered as statistically significant, respectively. All statistical analyses were carried out in *R* (4.0.4) (http://www.r-project.org/).

## Data availability

The data used in this study are not publicly available because they use clinical records protected by patient confidentiality. Requests for access to deidentified data can be directed to the corresponding author.

## Supporting information

This article contains supporting information

## Conflict of interest

The authors declare the following conflicts of interest with the contents of this article: at study start, J. N. and Fr. G. were listed as co-inventors in patent applications related to the biomarkers described in this study. At the time of submission, J. N. and Fr. G. were shareholders in Elypta AB, which owned the above-mentioned patent applications, J. N. was member of the board and Fr. G. was an employee in Elypta AB. All other authors declare no conflicts of interest.
